# Association between soluble suppression of tumorigenicity 2 and risk and severity of coronary artery disease: a case control study

**DOI:** 10.1186/s12872-025-04787-5

**Published:** 2025-04-28

**Authors:** Shuai Zhang, Lu Qian, Shibao Li, Zhijian Liu

**Affiliations:** 1 Department of Laboratory Medicine, Affiliated Hospital of Xuzhou Medical University, No.99 Huaihai West Road, Xuzhou, 221002 Jiangsu China; 2 Xuzhou Blood Center, Xuzhou, 221002 Jiangsu China

**Keywords:** Coronary artery disease, Soluble suppression of tumorigenicity 2, sST2, Inflammatory index

## Abstract

**Background:**

To investigate the differential expression of soluble suppression of tumorigenicity 2 (sST2) in patients with coronary artery disease (CAD) and healthy controls, and the correlation between sST2 and the severity of coronary artery atherosclerosis.

**Methods:**

A total of 911 CAD patients were selected as the CAD group, and 322 healthy people were selected as the control group. We measured serum sST2 level by chemiluminescence immunoassay, and applied the Gensini scoring system to quantify the severity of coronary artery atherosclerosis. We utilized Mann-Whitney U test to assess the difference of sST2 level between the two groups, and adopted Spearman correlation test to evaluate the correlation between sST2 level and Gensini score and inflammatory indexes.

**Results:**

Compared with the control group, the expression level of sST2 in CAD group was significantly increased [29.20 (20.67, 46.34) vs. 19.69 (15.97, 25.02), *P* < 0.001]. Logistic regression showed that sST2 expression could increase CAD risk (OR = 1.099, 95%CI: 1.080 ~ 1.119, *P* < 0.001). Analysis of variance revealed that the sST2 expression level increased gradually in unstable angina pectoris group (UA), non-ST elevation myocardial infarction group (NSTEMI) and ST elevation myocardial infarction group (STEMI) [UA: 23.05 (17.54, 30.75), NSTEMI: 30.71 (21.31, 42.97), STEMI: 51.05 (32.85, 80.04), *P* < 0.001]. Spearman correlation analysis demonstrated significantly positive associations between sST2 expression level and Gensini score (*r* = 0.137, *P* < 0.001), and systemic inflammatory indexes MHR (*r* = 0.188, *P* < 0.001), NLR (*r* = 0.469, *P* < 0.001), PLR (*r* = 0.285, *P* < 0.001) and MLR (*r* = 0.368, *P* < 0.001), but negatively correlated with AFR (*r*=-0.135, *P* < 0.001). By receiver operating characteristic (ROC) curve analysis, the sST2 expression level had excellent predictive effect in STEMI with the area under the curve (AUC) value of 0.926 (95%CI: 0.903–0.948, *P* < 0.001) and sensitivity and specificity of 72.3% and 99.7% respectively, superior to NSTEMI with an AUC of 0.760 (95%CI: 0.719–0.802, *P* < 0.001) and UA with an AUC of 0.616 (95%CI: 0.576–0.656, *P* < 0.001).

**Conclusions:**

sST2 could not only serve as a biomarker for the clinical auxiliary diagnosis of CAD, but also act as a potential indicator for disease progression or risk stratification. Dynamic monitoring of sST2 levels might assist in evaluating treatment efficacy.

## Introduction

Coronary artery disease (CAD) is the most common cardiovascular disease, with its incidence gradually increasing with age. In 2020, approximately 600 million people globally struggled with cardiovascular diseases, and approximately 19.05 million deaths were attributed to them, representing increases of 29.01% and 18.71% respectively compared to 2010 [1]. Based on data from the Atherosclerosis Risk in Communities Study (ARIC), it was estimated that approximately 605,000 new cases and 200,000 recurrent cases suffered from myocardial infarction annually. Among all the 805,000 myocardial infarction patients, about 170,000 cases existed without clinical symptoms [1]. According to the “Report on Cardiovascular Health and Diseases in China 2023: an Updated Summary”, 330 million people in China suffered from cardiovascular diseases, of which 11.39 million were CAD patients. Since 2012, the mortality rate of CAD has been on the rise, particularly notably in rural areas [2]. Pathobiological Determinants of Atherosclerosis in Youth (PDAY) study pointed out that 90% of coronary heart disease could be intercepted with the aid of early identification and proactive improvement of the major recognized risk factors, as well as the advocation of healthy lifestyle throughout the course of youth and adulthood [3].

The pathophysiology of CAD involves endothelial dysfunction, inflammatory activation, and lipid deposition, culminating in luminal stenosis or acute plaque rupture [4]. Early detection and accurate diagnosis are critical for mitigating adverse outcomes. Advances in diagnostic tools, ranging from non-invasive imaging modalities like coronary computed tomography angiography to invasive techniques such as coronary angiography, have led to the identification of both subclinical atherosclerosis and high-risk coronary lesions [5]. Emerging evidence suggests intriguing intersections between oncologic and cardiovascular pathophysiology. Several tumor-related parameters, including systemic inflammatory markers and interleukin-6 (IL-6), have been implicated in atherosclerotic progression [6]. Particularly, IL-33/ST2 signaling pathway is now recognized as a shared pathway in both tumor metastasis and plaque destabilization [7]. This biological duality positions ST2 as a unique biomarker bridging oncology and cardiology diagnosis.

Suppression of tumorigenicity 2 protein (ST2) is a protein produced by cardiomyocytes in response to biomechanical stress, existing mainly in two forms: soluble ST2 (sST2) and transmembrane ST2 (ST2L) [8]. The IL-33/ST2L signaling pathway plays cardiovascular protective role by antagonizing ventricular remodeling, reducing cardiomyocyte apoptosis, and improving cardiac function [9, 10]. By competitively binding with IL-33, sST2 can block the IL-33/ST2L signaling pathway and then involve in the entire process of atherosclerosis formation in CAD [8, 11]. Inflammatory biomarkers play a pivotal role in the pathogenesis and progression of atherosclerosis. Multiple inflammatory biomarkers, including high-sensitivity C-reactive protein (hs-CRP) and interleukin-6 (IL-6), not only reflect the inflammatory status of vascular walls but also predict plaque stability [6]. Notably, emerging research indicates that novel inflammatory markers such as neutrophil-to-lymphocyte ratio (NLR), monocyte-to-high-density lipoprotein ratio (MHR), platelet to lymphocyte ratio (PLR) and monocyte to lymphocyte ratio (MLR) demonstrate superior accuracy over traditional markers in evaluating plaque vulnerability [12].

Previous studies have primarily established the research advance of sST2 in the field of CAD, including its roles in reflecting atherosclerotic burden, predicting prognosis, reflecting myocardial remodeling, and guiding clinical management [13]. However, the association between sST2 and early diagnosis and disease progression of CAD remains under investigation. This study detected and analyzed the expression differences of sST2 in CAD patients and healthy controls, explored the associations of sST2 with systemic inflammatory indexes and the severity of CAD atherosclerosis and ultimately aimed to provide new insights for the diagnosis, treatment and prognosis of CAD.

## Materials and methods

### Study population

This study involved 911 CAD patients treated at the Affiliated Hospital of Xuzhou Medical University from January 2022 to July 2024 averaging an age of (64.51 ± 12.21) years, and 322 healthy individuals having a mean age of (62.98 ± 13.34) years. The diagnostic criteria for CAD were the presence of stenosis degree of more than 50% in left main and more than 70% in any other coronary artery or its main branches. CAD patients, included in the CAD group, must eliminate the following diseases: (1) cardiogenic diseases such as myocardial disease, pericardial disease, heart failure, valvular heart disease and et al.; (2) systemic diseases such as immunodeficiency disease, autoimmune diseases, benign and malignant tumors. The control group consisted of age and gender matched healthy individuals who underwent health check-ups during the corresponding period. Healthy people, designated to the control group, had normal blood glucose and lipid levels and excluded the aforementioned diseases through hematological examinations and imaging examinations. We also excluded CAD patients and healthy individuals with missing data. This study obtained ethical approval from the Medical Ethics Committee of the Affiliated Hospital of Xuzhou Medical University.

### Clinical data collection

We extracted the following clinical data: (1) Basic information: age, gender; (2) hematological examination data: fasting blood glucose, triglycerides, total cholesterol, low-density lipoprotein, high-density lipoprotein, blood monocyte count, lymphocyte count, platelet count, neutrophil count, fibrinogen, albumin; (3) Relevant medical history: history of alcohol drinking, smoking, hypertension and diabetes; (4) Coronary angiography report.

### Assessment of the severity of coronary atherosclerosis

We employed the Gensini scoring system, which consists of the weighting coefficient and the stenosis score, to evaluate the severity of coronary atherosclerosis [14]. The Gensini scoring system divides the coronary arteries into 15 segments and assigns different weighting coefficients to each segment. The degree of stenosis in each segment of the coronary artery is scored as follows: less than 25% stenosis is scored as 1, 25-49% as 2, 50-74% as 4, 75-89% as 8, 90-99% as 16, and 100% as 32. The final Gensini score is the cumulative sum of the product of each segment’s score and its corresponding weighting coefficient.

### Calculation of systemic inflammatory indexes

Based on the results of peripheral blood hematological examination, we calculated the following systemic inflammatory indexes: the Monocyte to High-density Lipoprotein Ratio (MHR), the Platelet to Lymphocyte Ratio (PLR), the Neutrophil to Lymphocyte Ratio (NLR), the Albumin to Fibrinogen Ratio (AFR) and the Monocyte to Lymphocyte Ratio (MLR).

### Detection of sST2 expression level in peripheral blood

We have collected peripheral blood from all included CAD patients and healthy control to isolate serum after centrifuging the coagulated blood at 4,000 r/min for 10 min. We used sST2 Assay Kit, purchased from Guangzhou Chunkang Biotechnology Co., Ltd., and the automated chemiluminescence analyzer, produced by Chongqing Keysmile Biological Technology Co., Ltd., to measure sST2 expression level in serum by chemiluminescence immunoassay.

### Statistical analyses

We used SPSS 26.0 software to conduct relevant statistical analyses. We also used the mean ± standard deviation (SD) and median with interquartile range (IQR) respectively to describe continuous variables that followed and failed to follow the normal distribution. We compared the differences of variables between two different groups by the Student’s t-test and Mann-Whitney U test. We employed the frequency with percentages and Pearson chi-square test to describe and analyze categorical variables respectively. We implemented the Spearman correlation test to evaluate the association between sST2 level and Gensini scores and inflammatory indexes. We used analysis of variance (ANOVA) to analyze the association between sST2 level and the clinical subtypes of CAD. Using the Youden index as a basis, we carried out receiver operating characteristic (ROC) curve to evaluate the predictive ability of sST2 in CAD by means of quantifying the area under the curve (AUC) and determining sensitivity, specificity and the most suitable cut-off values. Statistical significance was defined as a *P*-value below 0.05.

## Results

### Clinical characteristics of study population

This study included 911 CAD patients and 322 healthy individuals. There were no significantly differences between the CAD group and control group in age (64.51 ± 12.21 vs. 62.98 ± 13.34, *P* = 0.064) and gender (66.40% males vs. 66.46% males, *P* = 0.987). Compared to the control group, the CAD group had higher rates of smoking (31.10% vs. 13.04%, *P* < 0.001), hypertension (53.30% vs. 44.72%, *P* = 0.008) and diabetes (22.60% vs. 8.39%, *P* < 0.001), as well as higher neutrophil counts (5.82 ± 2.91 vs. 3.97 ± 2.03, *P* < 0.001) and monocyte counts (0.50 ± 0.24 vs. 0.41 ± 0.16, *P* < 0.001). The CAD group also exhibited significantly elevated levels of fasting blood glucose (6.33 ± 2.50 vs. 4.91 ± 0.58, *P* < 0.001), total cholesterol (4.25 ± 1.04 vs. 3.87 ± 0.88, *P* < 0.001), triglycerides (1.69 ± 1.12 vs. 1.14 ± 0.37, *P* < 0.001), low-density lipoprotein (2.60 ± 0.91 vs. 2.25 ± 0.73, *P* < 0.001), and fibrinogen (3.12 ± 0.95 vs. 2.81 ± 0.84, *P* < 0.001), along with lower levels of high-density lipoprotein (1.03 ± 0.28 vs. 1.10 ± 0.30, *P* < 0.001) and albumin (40.50 ± 4.47 vs. 41.92 ± 3.76, *P* < 0.001). No significant differences were observed in the rate of Alcohol drinking (15.10% vs. 10.87%, *P* = 0.057), lymphocyte count (1.58 ± 0.94 vs. 1.65 ± 0.57, *P* = 0.149) and platelet count (214.69 ± 62.11 vs. 212.90 ± 61.80, *P* = 0.657) between the two groups (Table 1).

**Table 1:** Clinical characteristics of study population

Variables	CAD group (*n* = 911)	Control group (*n* = 322)	*P*
Male [n (%)]	605(66.40)	214(66.46)	0.987
Age (years)	64.51 ± 12.21	62.98 ± 13.34	0.064
Smoking [n (%)]	274(30.10)	42(13.04)	< 0.001
Alcohol drinking [n (%)]	138(15.10)	35(10.87)	0.057
Hypertension [n (%)]	486(53.30)	144(44.72)	0.008
Diabetes [n (%)]	206(22.60)	27(8.39)	< 0.001
Fasting blood glucose (mmol/L)	6.33 ± 2.50	4.91 ± 0.58	< 0.001
Total cholesterol (mmol/L)	4.25 ± 1.04	3.87 ± 0.88	< 0.001
Triglycerides (mmol/L)	1.69 ± 1.12	1.14 ± 0.37	< 0.001
High-density lipoprotein (mmol/L)	1.03 ± 0.28	1.10 ± 0.30	< 0.001
Low-density lipoprotein (mmol/L)	2.60 ± 0.91	2.25 ± 0.73	< 0.001
Neutrophil count (10^9/L)	5.82 ± 2.91	3.97 ± 2.03	< 0.001
Lymphocyte count (10^9/L)	1.58 ± 0.94	1.65 ± 0.57	0.149
Monocyte count (10^9/L)	0.50 ± 0.24	0.41 ± 0.16	< 0.001
Platelet count (10^9/L)	214.69 ± 62.11	212.90 ± 61.80	0.657
Albumin (g/L)	40.50 ± 4.47	41.92 ± 3.76	< 0.001
Fibrinogen (g/L)	3.12 ± 0.95	2.81 ± 0.84	< 0.001

### Elevated expression of sST2 in the CAD group

**Fig. 1:**
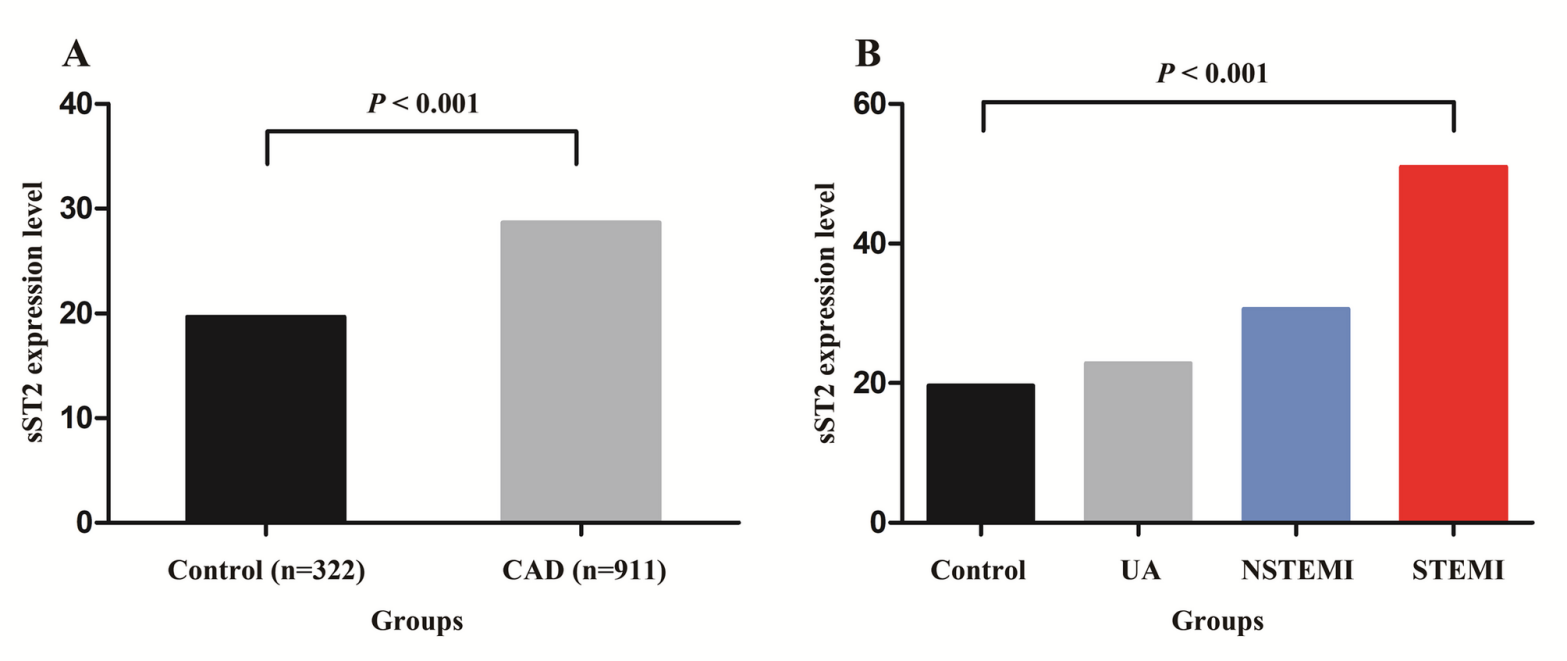
sST2 expression levels in different groups. (**A**). Comparison of sST2 expression levels between the control group and CAD group analyzed by Mann-Whitney U test. (**B**). Comparison of sST2 expression levels across the control group, unstable angina (UA) group, non-ST-segment elevation myocardial infarction (NSTEMI) group and ST-segment elevation myocardial infarction (STEMI) group analyzed by analysis of variance (ANOVA)

Compared to the control group, significantly elevated sST2 expression level was found in the CAD group [29.20 (20.67, 46.34) vs. 19.69 (15.97, 25.02), *P* < 0.001] (Fig. 1A). Logistic regression analysis revealed that elevated sST2 expression could increase the risk of CAD after adjustment for age, gender, history of smoking, alcohol drinking, hypertension, and diabetes (OR = 1.099, 95%CI: 1.080–1.119, *P* < 0.001). When stratified by clinical presentation, sST2 expression level showed progressive elevation across CAD subtypes: unstable angina (UA) [23.05 (17.54, 30.75)], non-ST-segment elevation myocardial infarction (NSTEMI) [30.71 (21.31, 42.97)] and ST-segment elevation myocardial infarction (STEMI) [51.05 (32.85, 80.04)], with significant differences identified through ANOVA (*P* < 0.001) (Fig. 1B).

### Associations between sST2 level and the severity of coronary atherosclerosis

**Fig. 2:**
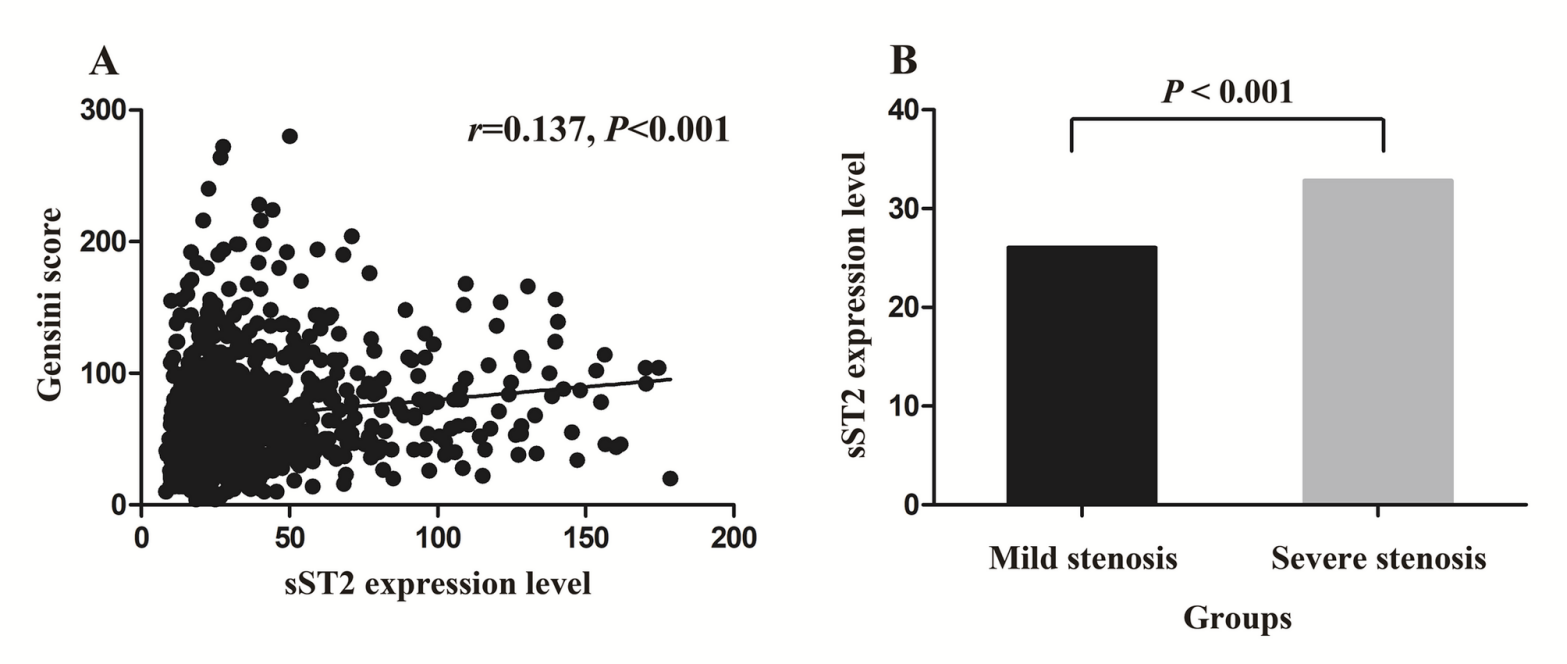
Expression and association of sST2. (**A**). Correlation of sST2 expression level with the Gensini score analyzed by Spearman correlation test. (**B**). Comparison of sST2 expression levels between the mild and severe stenosis group analyzed by Mann-Whitney U test

Spearman correlation analysis, conducted on the sST2 expression level and Gensini score in the CAD group, showed a positive correlation between sST2 expression level and Gensini scores (*r* = 0.137, *P* < 0.001) (Fig. 2A). Using the median of Gensini score as the threshold value, CAD patients were divided into the severe stenosis group (Gensini score > 58) and the mild stenosis group (Gensini score ≤ 58). Mann-Whitney U test analysis revealed that the severe stenosis group exhibited significantly greater sST2 expression level than the mild stenosis group [32.85 (22.37, 52.32) vs. 26.09 (19.34, 39.64), *P* < 0.001] (Fig. 2B), further confirming the association between sST2 expression level and the severity of coronary atherosclerosis.

### Correlations of sST2 expression level with risk factors for CAD

Correlation analysis on the combined data of the CAD group and the control group showed that sST2 expression level was significantly associated with age (*r* = 0.050, *P* = 0.04), gender (*r* = 0.110, *P* < 0.001), smoking (*r* = 0.114, *P* < 0.001), and diabetes (*r* = 0.069, *P* = 0.016). Subsequently, the combined data were divided into two subgroups based on the average age, gender, history of smoking and history of diabetes. The Mann-Whitney U test demonstrated significant differences of sST2 expression levels between the two subgroups, which also indicated that the sST2 expression level was associated with age, gender, smoking, and diabetes (Table 2).

**Table 2:** The expression difference of sST2 in subgroups [ M (P25, P75)]

Variables	Subgroups	sST2 expression level	*P*
Age (years)	>64	26.96(20.08,39.22)	<0.001
	≤ 64	24.41(17.50,36.40)	
Gender	Male	27.72(20.49,39.88)	<0.001
	Female	21.31(15.97,31.53)	
Smoking	Yes	29.00(20.61,47.17)	<0.001
	No	24.53(18.20,35.63)	
Diabetes	Yes	27.75(20.47,44.56)	0.001
	No	24.86(18.37,36.51)	

### Correlations between sST2 expression level and inflammatory indexes

**Fig. 3:**
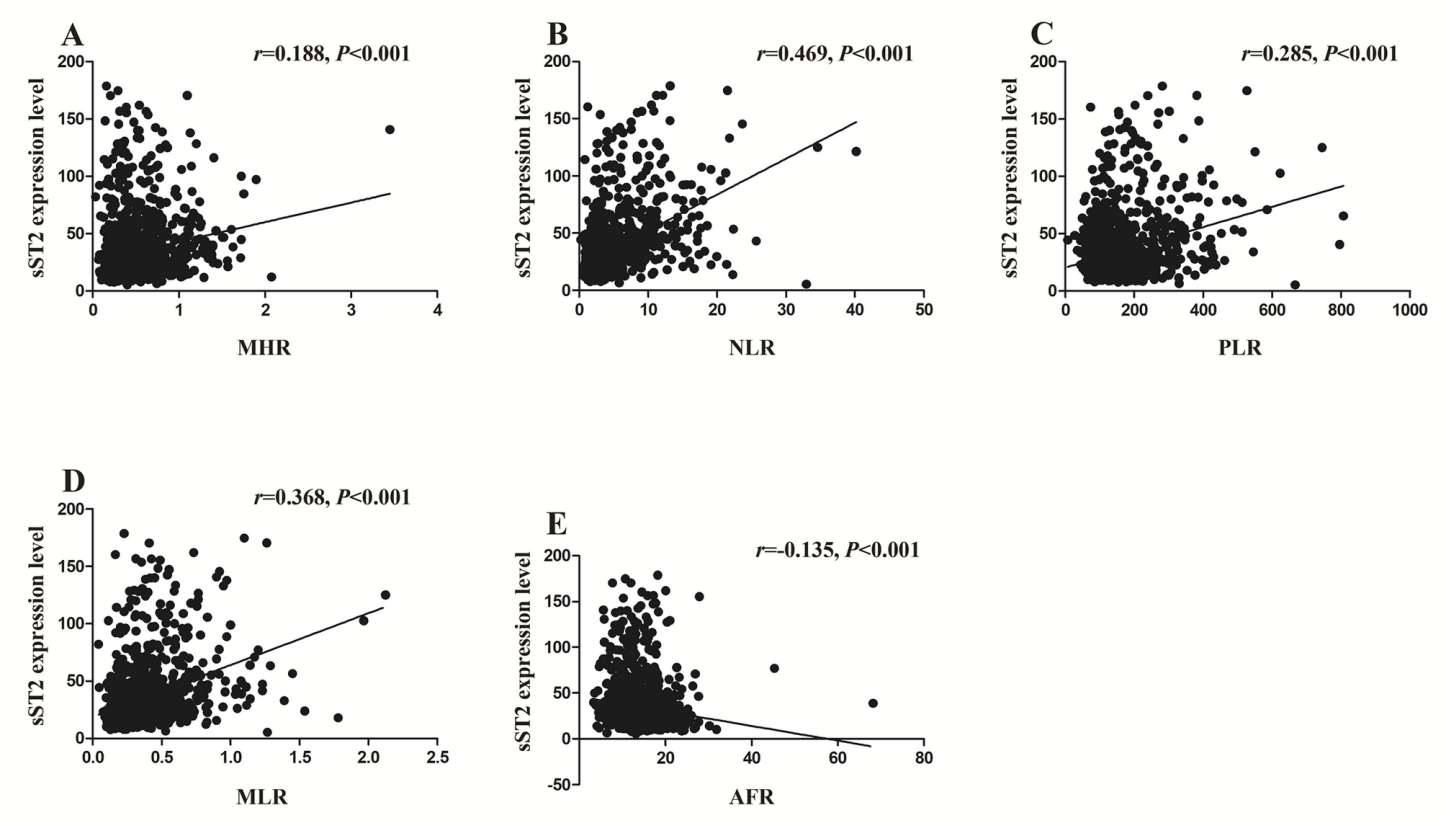
Correlations of sST2 expression level with inflammatory indexes analyzed by Spearman correlation test. sST2 expression level was positively correlated with the Monocyte to High-density Lipoprotein Ratio (MHR, *r* = 0.188, **A**), the Neutrophil to Lymphocyte Ratio (NLR, *r* = 0.469, **B**), the Platelet to Lymphocyte Ratio (PLR, *r* = 0.285, **C**), the Monocyte to Lymphocyte Ratio (MLR, *r* = 0.368, **D**), and negatively correlated with the Albumin to Fibrinogen Ratio (AFR, *r*=-0.135, **E**)

Spearman correlation analyses revealed that the sST2 expression level was positively related to MHR (*r* = 0.188, *P* < 0.001), NLR (*r* = 0.469, *P* < 0.001), PLR (*r* = 0.285, *P* < 0.001), and MLR (*r* = 0.368, *P* < 0.001), while it was inversely associated with AFR (*r* = − 0.135, *P* < 0.001) (Fig. 3).

### ROC analyses to evaluate the efficiency of sST2 in predicting CAD and its clinical subtypes

**Fig. 4:**
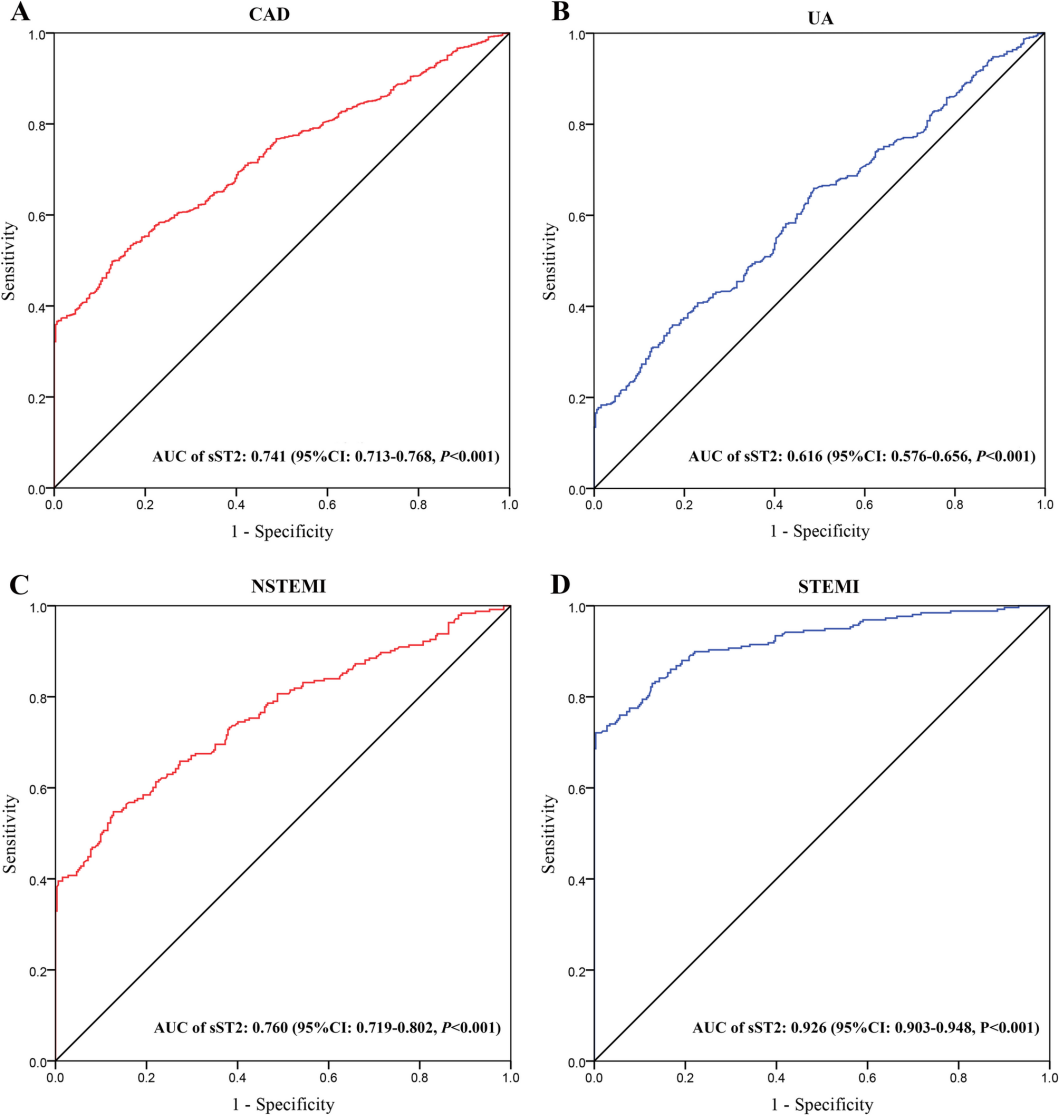
Receiver operating characteristic (ROC) curve analyses of sST2 in predicting coronary artery disease (CAD) (**A**), unstable angina (UA) (**B**), non-ST-segment elevation myocardial infarction (NSTEMI) (**C**) and ST-segment elevation myocardial infarction (STEMI) (**D**)

By ROC curve analysis, the area under the curve (AUC) of sST2 to predicting CAD was 0.741 (95%CI: 0.713–0.768, *P* < 0.001) with a sensitivity of 51.7% and a specificity of 87.3% (Fig. 4; Table 3). Subsequently, we carried out the ROC curve analyses in clinical subtypes of CAD. Subjects with sST2 expression level exceeding the cut-off value of 35.02 were more likely to have higher risk of STEMI, along with statistically significant sensitivity and specificity of 72.3% and 99.7% respectively (Fig. 4; Table 3). The sST2 expression level had excellent predictive effect in STEMI with an AUC of 0.926 (95%CI: 0.903–0.948, *P* < 0.001), superior to NSTEMI with an AUC of 0.760 (95%CI: 0.719–0.802, *P* < 0.001) and UA with an AUC of 0.616 (95%CI: 0.576–0.656, *P* < 0.001) (Fig. 4; Table 3).

**Table 3:** Evaluations of the efficiency of sST2 in predicting CAD and its clinical subtypes

Groups	AUC	95%CI	*P*	Sensitivity	Specificity	Cut-off value
CAD	0.741	0.713 ~ 0.768	< 0.001	0.517	0.873	28.85
UA	0.616	0.576 ~ 0.656	< 0.001	0.358	0.816	27.04
NSTEMI	0.760	0.719 ~ 0.802	< 0.001	0.552	0.873	28.88
STEMI	0.926	0.903 ~ 0.948	< 0.001	0.723	0.997	35.02

AUC: area under the curve; CAD: coronary artery disease; UA: unstable angina; NSTEMI: non-ST-segment elevation myocardial infarction; STEMI: ST-segment elevation myocardial infarction; CI: confidence interval

## Discussion

CAD is a type of disease that atherosclerotic alterations occurring in the coronary arteries and lipids from the blood depositing on the intima of the arteries, and then leading to the stenosis or obstruction of the vessel lumen, and ultimately causing myocardial ischemia or necrosis [15]. The principal pathological change of CAD is atherosclerosis, which is a chronic inflammatory disease of the arteries related to various factors, including cardiovascular health factors such as advanced age, smoking, alcohol drinking and obesity, as well as cardiovascular risk factors such as hypertension, diabetes and dyslipidemia [16]. Due to the widespread prevalence of unhealthy dietary habits, insufficient physical activity, the sharp increase of smokers and alcohol drinkers, and the acceleration of population aging, individuals with cardiovascular risk factors in our country is still huge, and the rates of CAD-related illness and death are steadily growing [16]. Investigating the role of genetic and environmental factors in the occurrence and development of CAD contributes to improving the prevention and treatment of CAD.

As the specific ligand for ST2, IL-33 binds to ST2L and activates downstream mitogen-activated protein kinases (MAPK) and nuclear factors-κB (NF-κB) of the IL-33/ST2L signaling pathway when cardiomyocytes and fibroblasts suffer mechanical stimulation or damage [17]. These events have the potential to play cardioprotective effects by reducing myocardial fibrosis, preventing cardiomyocyte hypertrophy, decreasing cardiomyocyte apoptosis and promoting myocardial function [11, 18]. Acting as a decoy receptor, sST2 could competitively bind to IL-33 and block the IL-33/ST2L signaling pathway, thereby leading to the loss of its cellular regulatory functions [19]. Studies have shown that serum sST2 levels rose significantly in the early stage of myocardial infarction and were related to left ventricular ejection fraction and cardiac remodeling after myocardial infarction, which had the potential to be used for indicators for predicting mortality and prognosis of acute myocardial infarction [20, 21]. Compared to wild-type mice, targeted ST2-disrupted mice exhibited more severe left ventricular hypertrophy and myocardial fibrosis, as well as the loss of the IL-33 function [22]. Our investigation also found that significantly elevated expression level of sST2 in the CAD patients could increase the risk of CAD. Therefore, sST2 might participate in the occurrence and development of CAD through the IL-33/ST2L signaling pathway.

The present study has uncovered a direct relationship between sST2 expression levels and Gensini scores, and that the severe stenosis group exhibited significantly greater sST2 expression level than the mild stenosis group. Elevated sST2 levels have been shown to directly correlate with higher Gensini scores, indicating a potential role of sST2 as a biomarker for the severity of CAD [23]. Recent study demonstrated that elevated sST2 levels were significantly associated with increased disease severity and mortality risk in cardiovascular diseases. In patients with chronic heart failure and stable coronary artery disease (CAD), higher sST2 levels correlated with poorer clinical outcomes. This correlation suggested that sST2 might counteract the protective effects of the IL-33/ST2L pathway by acting as a decoy receptor, thereby contributing to adverse cardiovascular outcomes [24]. Earlier studies have indicated that sST2 expression levels in the UA patients were observably higher than that in the control group, but significantly lower than that in NSTEMI patients. Moreover, sST2 expression levels in the NSTEMI patients significantly decreased compared to the STEMI patients [25, 26]. This study also discovered that sST2 expression levels gradually increased in the UA group, NSTEMI group and STEMI group, with statistically significant differences. This progressive increase further demonstrated that sST2 expression level was connected with the severity of coronary atherosclerosis, and could serve as a serological biomarker reflecting the severity of myocardial injury and the intensity of inflammatory response.

In the present study, sST2 expression level was confirmed to be correlated with various systemic inflammatory indexes. Research by Selvaggio et al. shown that increased levels of MHR, NLR, and PLR were associated with peripheral arterial disease, which is one type of atherosclerotic cardiovascular disease [27]. Compared to the control group, MHR levels were markedly elevated in STEMI patients, and were associated with poor prognosis of acute coronary syndrome [28]. With the assistance of the class A scavenger receptors (SR-A) and CD-36, monocytes could absorb oxidized low-density lipoprotein and differentiate into foam cells, which has played a crucial role in the initial stage of atherosclerosis formation [29]. High-density lipoprotein had the potential to inhibit the activation and function of monocytes, promote the remove of cholesterol in macrophages, and then suppress the oxidation of low-density lipoprotein [30]. Significantly increased levels of MLR and NLR in the CAD patients were related to the extent of coronary atherosclerotic lesions, which could function as a tool for assessing coronary atherosclerotic plaque burden, and besides serving as independent predictive factors for CAD [31, 32]. When it was activated, neutrophils secreted various granular proteins and matrix-degrading proteases, induced the adhesion and aggregation of monocytes, and then aggravated endothelial cell apoptosis and dysfunction, and ultimately accelerated foam cell formation and the rupture of fibrous cap [33]. In our study, a stronger association was found between sST2 and NLR compared to PLR and MLR, which might arise from the cell-specific roles of the IL-33/ST2 pathway. As a decoy receptor for IL-33, sST2 primarily modulates inflammation by suppressing Th2 immune responses. Neutrophils, as central players in acute inflammation, likely exhibit count changes more directly tied to sST2-mediated Th2 immune modulation [34]. Monocytes usually play an important role in the later stages of inflammation and their activation and recruitment are influenced by different cytokines and pathways [35]. In addition, platelet counts are influenced by diverse non-inflammatory factors, including coagulation and endothelial function, attenuating the correlation of sST2 with PLR [36]. Methodologically, broader dynamic range of NLR may better capture sST2-associated inflammatory fluctuations. Fibrinogen could participate in platelet activation and aggregation, induce the injury, migration and dysfunction of vascular endothelial cell, promote vascular smooth muscle cell proliferation and migration into the subendothelial layer. In addition, fibrinogen had the potential to promote low-density lipoprotein oxidation, and subsequently lead to the formation of atherosclerotic plaque through vascular inflammatory responses [37]. Duan et al. also found that significantly decreased levels of AFR expression in the acute coronary syndrome patients were correlated to the extent of the lesion, which could serve as a novel biomarker for forecasting the onset and severity of CAD [38]. The aforementioned inflammatory indexes could suggest more sensitively than a single indicator that sST2 might participate in the occurrence and development of CAD through inflammatory responses and reflect the severity and prognosis of CAD.

ROC curve analyses revealed an excellent predictive effect of sST2 expression level in STEMI (AUC = 0.926), superior to NSTEMI (AUC = 0.760) and UA (AUC = 0.616). The striking AUC gradient (STEMI > NSTEMI > UA) strongly suggests that sST2 release is proportional to the extent of myocardial injury. STEMI is typically caused by complete coronary artery occlusion, leading to acute myocardial ischemia and necrosis. In this scenario, the inflammatory response and myocardial injury are more severe [39]. In contrast, the pathophysiology of NSTEMI and UA involve partial vessel occlusion or unstable plaque rupture, with a less severe inflammatory response [40]. The transient ischemia in UA fails to fully activate the IL-33/ST2 signaling pathway, resulting in relatively weaker predictive power of sST2 in these conditions. These findings argue against using sST2 as an independent diagnostic tool for UA, but support its integration into STEMI diagnostic pathway and post-MI risk stratification.

In light of these findings, we acknowledge several limitations and suggest directions for future research. First, the monocentric design and case–control study design may limit the generalizability of our findings and restrict our ability to infer causality. We plan to overcome these limitations in future studies by adopting a multicentric study design and prospective cohort study to more accurately assess the relationship between sST2 and CAD. Second, our study focused on diagnostic value and severity assessment of sST2, and did not evaluate its predictive value for long-term clinical outcomes such as mortality or recurrent cardiovascular events. Future prospective studies with extended follow-up and prognostic analyses are needed to fully understand its clinical utility in CAD management. Third, although we have adjusted for common cardiovascular risk factors in our study, residual confounding factors from unmeasured variables might still exist. Future studies should consider a broader range of potential confounding factors, including detailed comorbidities and medication use, to more accurately assess the clinical significance of sST2. Fourth, the relatively low AUC value fails to support sST2 as an independent diagnostic tool for UA. Future research should focus on identifying more suitable biomarkers or developing comprehensive assessment methods to improve the diagnosis and prognosis evaluation in UA patients.

In conclusion, sST2 could not only serve as a biomarker for the clinical auxiliary diagnosis of CAD, but also act as a potential indicator for disease progression or risk stratification. Dynamic monitoring of sST2 levels might assist in evaluating treatment efficacy. Based on the observed predictive value, we suggest that routine sST2 testing should be considered for all suspected CAD patients at hospital admission, especially STEMI and NSTEMI patients. Particular attention should be paid to patients with sST2 levels above 35.02, as these may benefit from dynamic monitoring and therapy. However, the results should be interpreted in conjunction with established biomarkers and clinical assessment before sufficient validations in prospective multicenter studies.

## Data Availability

The datasets used and/or analyzed during the current study are available from the corresponding author on reasonable request.

## Abbreviations

sST2: Soluble suppression of tumorigenicity 2
ST2L: Transmembrane suppression of tumorigenicity 2
CAD: Coronary artery disease
UA: Unstable angina pectoris
NSTEMI: Non-ST elevation myocardial infarction
STEMI: ST elevation myocardial infarction
MHR: Monocyte to High-density Lipoprotein Ratio
MLR: Monocyte to Lymphocyte Ratio
NLR: Neutrophil to Lymphocyte Ratio
AFR: Albumin to Fibrinogen Ratio
PLR: Platelet to Lymphocyte Ratio
MAPK: Mitogen-activated protein kinases
NF-κB: Nuclear factors-κB
ANOVA: Analysis of variance
IQR: Interquartile range
SR-A: Class A scavenger receptors
